# Clinico-Radiological Outcomes of Arthroscopic Rotator Cuff Repair With a Single-Row Construct Supplemented With Bone Marrow Vents

**DOI:** 10.7759/cureus.100261

**Published:** 2025-12-28

**Authors:** Deepak K Sharma, Anurag Sharma, Anmol Kankane, Anant Kumar, Narendra Kumar

**Affiliations:** 1 Central Institute of Orthopaedics, Vardhman Mahavir Medical College and Safdarjung Hospital, New Delhi, IND

**Keywords:** arthroscopic rotator cuff repair, arthroscopic shoulder surgery, bone marrow vents, orthopedic sports medicine, single-row repair, tendon healing

## Abstract

Introduction: Full-thickness rotator cuff tears are common in aging populations and pose healing challenges and significant re-tear rates due to a variety of factors, especially in large or massive tears. Arthroscopic single-row repair construct, though simpler and cost-effective, has biomechanical limitations. Adding bone marrow vents may improve biological healing via the release of certain growth factors through those vents. This study evaluates the outcomes of this combined approach and factors influencing tendon recovery.

Methods: This prospective study involved 22 patients with full-thickness rotator cuff tears that were confirmed on magnetic resonance imaging (MRI) scans and were unresponsive to conservative treatment modalities. Patients underwent arthroscopic single-row repair along with vents created over the greater tuberosity adjacent and lateral to the anatomical footprint using a micro-fracture awl. Outcomes of 21 patients were assessed using the American Shoulder and Elbow Surgeons (ASES) scores at three months and six months compared with the preoperative score. A follow-up MRI at six months was done to evaluate clinical and radiological healing. One patient was lost to follow-up.

Results: A total of 21 patients were available for follow-up, and a repeat MRI was then performed after a minimum duration of six months. All patients showed significant clinical improvement in the ASES scores. MRI confirmed tendon healing in 20 (95.2%) cases. No demographic or lifestyle factors significantly affected the outcomes. One (4.7%) case developed adhesive capsulitis in the postoperative period that resolved conservatively with medications and physiotherapy, indicating an overall positive result.

Discussion: Our study shows that arthroscopic single-row rotator cuff repair augmented with bone marrow vents offers enhanced outcomes for medium to large rotator cuff tears. Single-row repair has been considered an inferior construct as compared to double-row repair, but augmenting the single row with bone marrow vents has proven to show decreased re-tear rates in multiple studies. While trends suggest younger age and traumatic etiology may favor healing, a statistically significant correlation was limited by the small sample size and a shorter duration of follow-up.

Conclusion: Single-row repair of the rotator cuff with bone marrow vents enhances the biological healing effectively. Significant functional improvement indicated with improved ASES score and high tendon healing rates on a follow-up MRI support its use as a reliable and cost-effective technique to gain desirable outcomes in patients. Larger studies are needed to confirm the role of clinical factors that influence long-term outcomes, but our findings have proved to be promising and clinically relevant.

## Introduction

Rotator cuff tears constitute a frequent source of shoulder pain, particularly within an aging population. The management strategies for these tears have undergone significant advancements over recent decades, notably due to the introduction of arthroscopic techniques. Multiple surgical approaches have emerged, including arthroscopic single-row and double-row rotator cuff repairs. However, the ongoing discourse regarding the superiority of these methods reveals that no clear advantage exists in all cases. The single-row repair technique is frequently regarded as a cost-effective option; however, it is accompanied by several limitations, including diminished footprint coverage, reduced bio-mechanical strength, elevated re-tear rates, and a smaller tendon-to-bone contact area [1]. Conversely, the double-row rotator cuff repair does offer mechanical advantages. Still, it presents its own challenges, such as a protracted learning curve, increased costs, a heightened risk of over-tensioning the tendon (which may compromise blood supply and healing), a greater likelihood of shoulder stiffness, and more extensive manipulation of soft tissues. The single-row repair has garnered attention for its simplicity and effectiveness among the range of surgical techniques available. When employed in conjunction with the technique of bone marrow venting, an approach that facilitates biological augmentation by promoting healing factors from the bone, this method demonstrates promise in enhancing tendon healing and improving clinical results. Nevertheless, achieving consistent radiological healing for medium to large full-thickness rotator cuff tears remains a significant challenge, with reported re-tear rates varying between 20% and 94% [1]. A variety of factors may influence healing rates. Patient-related factors include advanced age, chronicity of symptoms, co-morbidities, fatty degeneration, and the size of the tear. Additionally, surgeon-related factors encompass the type of surgical construct utilized and the tension applied to the muscle-tendon unit during the repair [1].

Arthroscopic rotator cuff repair has been widely reported to produce favorable subjective and functional outcomes. However, the success of tendon healing following surgery varies greatly depending on the size of the tear. While small tears show healing rates of up to 90%, this rate decreases to around 30% for large tears and may fall as low as 6% in the case of massive tears. The variability in healing is also influenced by the biological quality of the rotator cuff tissues. Poor bone quality, tendon degeneration, and muscle atrophy or fatty infiltration are frequently cited as major factors contributing to tendon non-healing or re-tears [2].

One of the key challenges in rotator cuff repair is the avascular and atrophic nature of the torn tendon edges, which compromises their capacity for healing. Tendon-to-bone healing after repair largely depends on cellular proliferation and revascularization, with new blood vessels primarily originating from peri-bursal soft tissues after debridement, and adjacent bone at the footprint being prepared to promote bleeding. However, Snyder and Burns [3] argue that these methods may not be sufficient to provide the necessary vascular supply for optimal healing. They recommend the use of marrow venting or micro-fracture techniques, creating deeper perforations in the greater tuberosity, which may allow the release of bone marrow-derived elements such as stem cells, growth factors, and cytokines. These components can enhance the biological environment at the repair site, potentially improving tendon healing and reducing re-tear rates.

Innovative strategies for enhancing tendon healing encompass the use of growth factors, cytokines, gene therapy, and tissue engineering. However, the effectiveness of these approaches remains under evaluation. Recent biomechanical research has concentrated on refining repair techniques to boost the mechanical strength of rotator cuff tendon repairs and improve overall healing outcomes.

This study aims to assess the clinical and radiological outcomes of patients with complete rotator cuff tears who underwent arthroscopic single-row repair combined with bone marrow vents. Since we were looking for a technique that strikes a balance between the technical simplicity of single-row repair and the biological advantages associated with double-row repair, we chose to incorporate bone marrow vents into the single-row approach. In addition to evaluating outcomes, the study also seeks to identify factors that may influence healing and overall patient recovery.

## Materials and methods

A prospective, observational study was conducted at a tertiary care center in New Delhi, India. The study included 22 patients between the ages of 18 and 80 years. One patient out of 22 was eventually lost to follow-up. Surgery was considered for these patients as they exhibited significant pain and functional limitations that did not improve with conservative treatments such as physiotherapy, activity modification, and medication. A full-thickness tear of the supraspinatus and/or infraspinatus tendons confirmed by a magnetic resonance imaging (MRI) scan of the shoulder was a prerequisite for the procedure.

Patients with a prior history of shoulder surgery, cervical spine pathology, low-grade partial rotator cuff tears, systemic connective tissue diseases, massive irreparable rotator cuff tears, isolated subscapularis tears, Goutallier grade 3 and above fatty degeneration, and patients who did not give consent for the study were excluded from the study.

Patient demographic data and history of substance use (tobacco/alcohol) were recorded at the initial visit. Clinical outcomes were assessed using the American Shoulder and Elbow Surgeons (ASES) questionnaire at baseline (preoperative), followed by three months and six months postoperatively [4]. “Clinical improvement” was defined as an increase in the baseline ASES score within six months, whereas patients whose scores remained unchanged or declined were classified as having experienced “clinical failure.”

MRI was performed at baseline (not more than three months before the surgery), and the scans were reviewed by a trained musculoskeletal radiologist who was blinded to the patient's clinical outcome. The criteria given by Sugaya et al. [5] were used to assess tendon healing at the end of six months (Table 1). Patients were divided into two groups based on this classification. Treatment was considered “radiological success” if the tear had healed (Sugaya types I, II, and III), whereas a discontinuity in the tendon on more than one slice was classified as “radiological failure” (Sugaya types IV and V).

**Table 1:** Classification of rotator cuff repair healing based on postoperative MRI. Classification was given by Sugaya et al. [5].

Types	Radiological (MRI) findings
Type I	Sufficient thickness, homogenous tendon (low signal on T2 images)
Type II	Sufficient thickness, partial high-intensity signal from within the tendon
Type III	Insufficient thickness (<50%) without discontinuity
Type IV	Minor discontinuity on more than one slice, suggesting a small tear
Type V	Major discontinuity suggesting a moderate or large tear

Surgical technique

**Figure 1:**
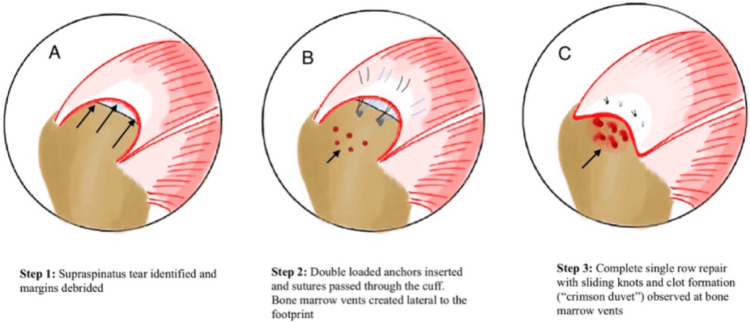
Schematic diagram showing retracted cuff tissue with arrows in panel A and the stepwise technique of single-row arthroscopic rotator cuff repair. Bone marrow vents marked with arrows are seen in panel B, and a “crimson duvet” through those vents in panel C. The figure is the original artwork created by the authors.

All patients were given general anesthesia along with an interscalene block. Patients were positioned in the lateral decubitus position with a longitudinal and vertical two-pulley traction system, with 40-50° abduction, and kept in a neutral position. Standard posterior, lateral, and anterior portals were made, and diagnostic arthroscopy of the glenohumeral and subacromial space was done, and the associated pathologies were treated, such as subacromial decompression for mechanical abrasion of the coracoacromial ligament. The tendon was shaved to achieve a stable edge, and the anatomic footprint of the tendon, as well as the size of the tear, was measured. The repair was done based on the size and pattern of the tear. Double-loaded suture anchors made up of all-suture, polyether ether ketone (PEEK), or metal anchors were used for the repair, selected according to the patient’s profile and affordability. A suture anchor was inserted into the bone at the "Deadman angle." The anchors were placed at nearly 5 mm distance from the articular cartilage to decrease tension at the tendon, and bites were taken through the cuff tissue using suture passing devices. Bone marrow vents were created using a straight bone awl over to the greater tuberosity adjacent to the anatomical footprint at a distance of 3 to 5 mm from each other. A schematic diagram showing a stepwise technique can be seen in Figure 1.

**Figure 2:**
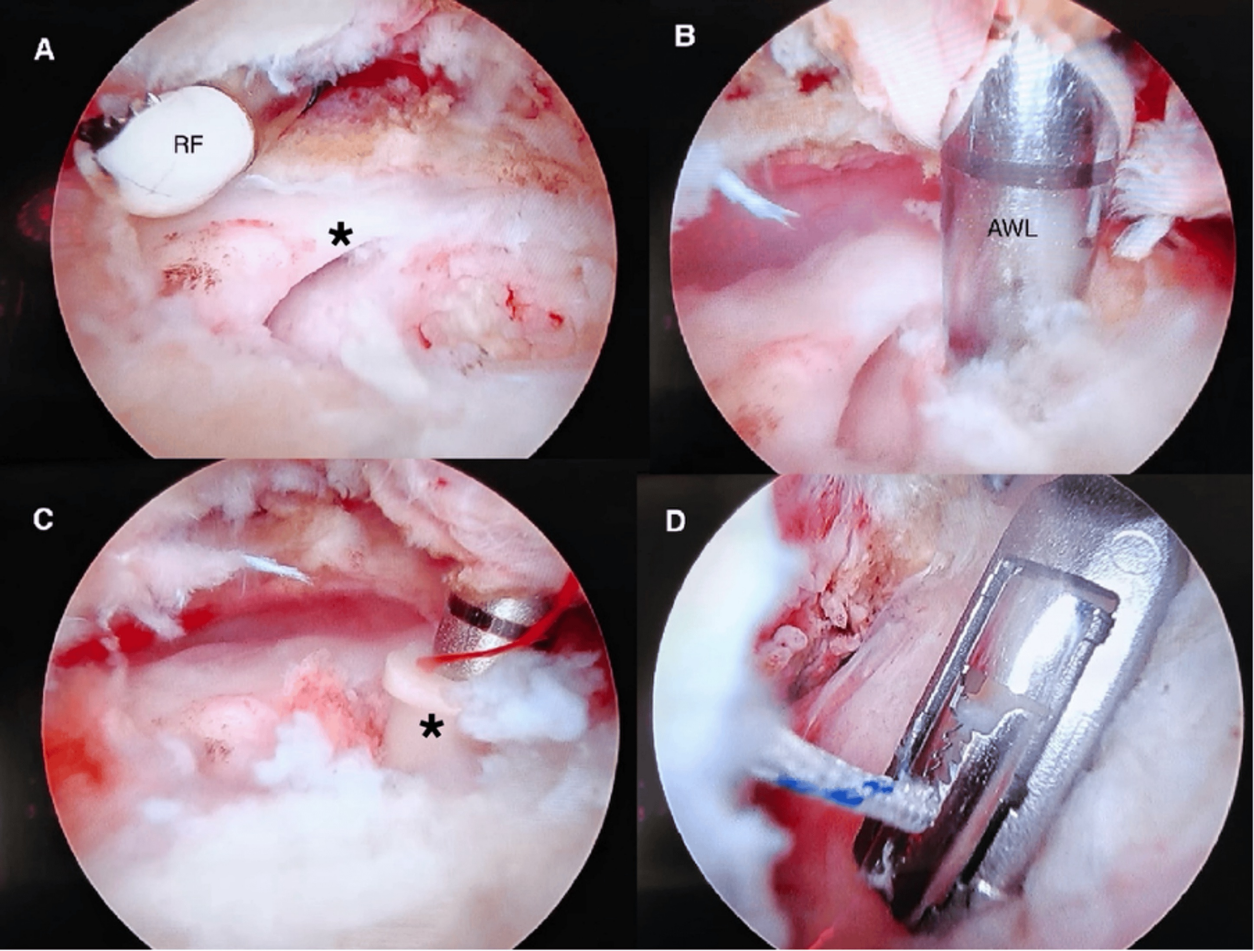
Arthroscopic images showing a stepwise approach to the procedure. (A) Identification of the rotator cuff tissue (asterisk) with edges debrided using a radiofrequency (RF) probe. (B) Placing an entry awl for the suture anchor at the Deadman’s angle. (C) Placement of the suture anchor (asterisk). (D) Suture passed through the cuff tissue using a suture passing device.

**Figure 3:**
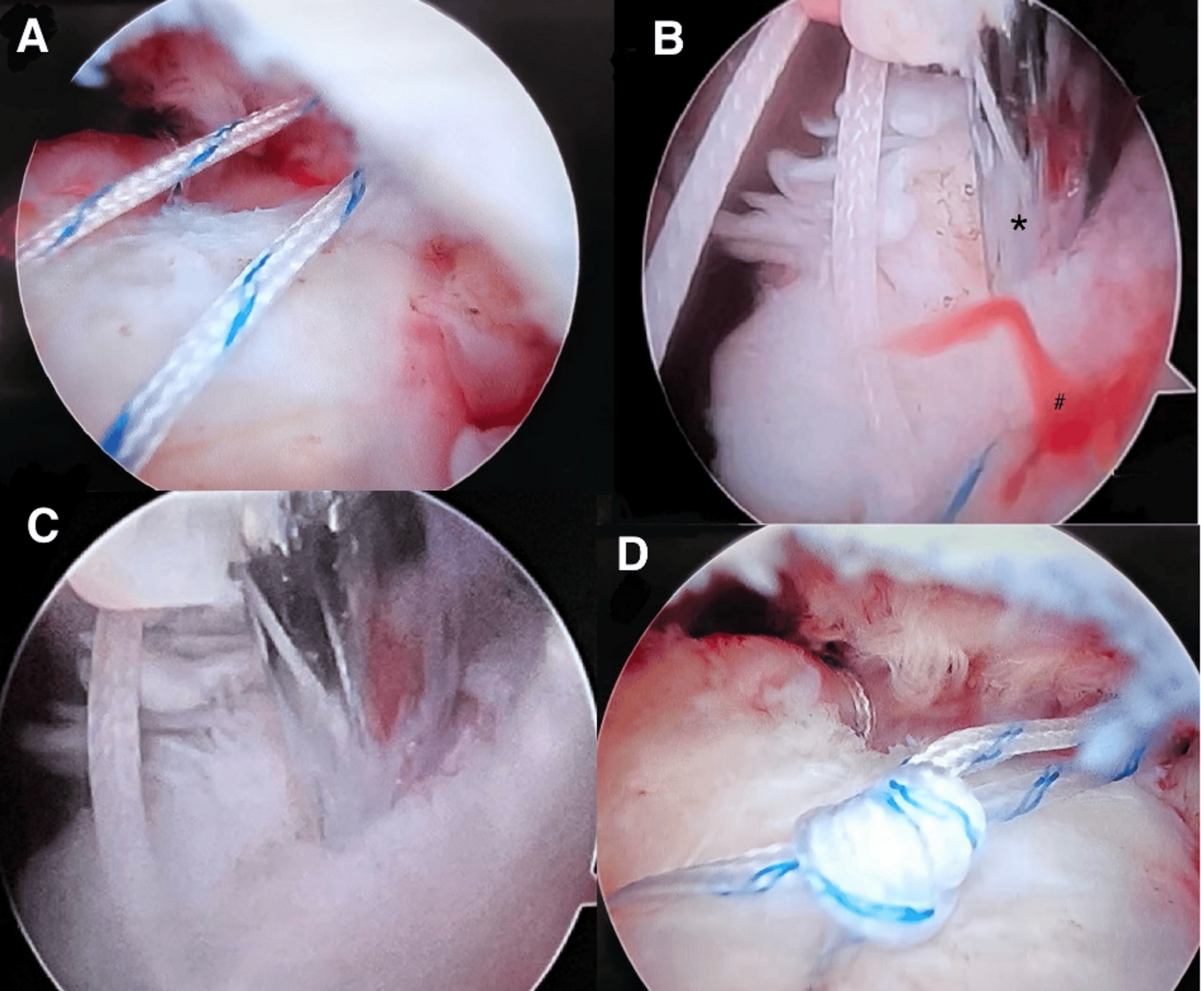
Arthroscopic images showing a stepwise approach to the procedure. (A) Passed sutures through the cuff. (B) Microfracture awl (asterisk) used to create a bone marrow vent and visualized “crimson duvet” (#) through the vent. (C) Another bone marrow vent was created using a microfracture awl. (D) Sliding knot with completed single-row repair.

Release of pluripotent bone marrow stem cells and growth factors was anticipated through these bone marrow vents. Biological healing via growth factors, such as vascular endothelial growth factor (VEGF) and platelet-derived growth factors (PDGF), as well as stem cells, augments the mechanical healing. Following placement of sutures through the tendons, they were reduced and secured using a sliding knot and three alternatively reversed half-hitches. Clot formation, aka “crimson duvet” [6], was observed over the bone marrow vents. Portals were closed with surgical staples, and an aseptic dressing was applied. Arthroscopic images describing the steps of the procedure can be seen in Figures 2, 3.

Postoperatively, the patients were immobilized in a universal shoulder immobilizer in neutral rotation and abduction for four weeks. All patients followed standard institutional protocol for physiotherapy and rehabilitation. Elbow, wrist, and hand exercises were started on postoperative day one, pendulum exercises at two weeks, active assisted forward flexion and abduction at four weeks, and strengthening exercises were started at eight weeks. Patients were allowed full activity at 20 weeks.

Statistical analysis

The data were analyzed using SPSS version 21.0 (IBM Corp., Armonk, NY). Chi-square test or Fisher's exact test was used for categorical data, whereas the Mann-Whitney U test was used for quantitative data. The Friedman test was used to make statistical inferences where the data were not normally distributed. A p-value of <0.05 was considered statistically significant.

## Results

A total of 22 patients were initially enrolled in this prospective study over 12 months. Of these, 21 patients were available for final analysis at the six-month follow-up, with one patient lost to follow-up. The age of the participants ranged from 32 to 74 years, with a mean age of 55.18 ± 12.86 years, representing a wide distribution across middle-aged and older adults, the demographic most commonly affected by rotator cuff pathology.

Of the total sample, 13 (59.1%) patients were male, and nine (40.9%) were female, reflecting a slight male predominance consistent with broader epidemiological data on rotator cuff injuries. The dominant shoulder was involved in 16 (72.7%) patients, suggesting a potential relationship between repetitive use and the development of tears. The remaining six (27.3%) patients had injuries affecting the non-dominant shoulder (Table 2).

**Table 2:** Demographic characteristics of the participants.

Basic details	Categories	Number of patients (%)
Age	31-40 years	3 (13.6%)
	41-50 years	6 (27.3%)
	51-60 years	3 (13.6%)
	61-70 years	7 (31.8%)
	71-80 years	3 (13.6%)
Gender	Male	13 (59.1%)
	Female	9 (40.9%)
Side	Dominant	16 (72.7%)
	Non-dominant	6 (27.3%)
Etiology	Traumatic	18 (81.8%)
	Non-traumatic	4 (18.2%)
Alcohol use	Alcoholic	4 (18.2%)
	Non-alcoholic	18 (81.8%)
Smoking	Smoker	5 (22.7%)
	Non-smoker	17 (77.3%)

Regarding etiology based on history obtained, the majority of tears, i.e., 18 (81.8%) patients, were of traumatic origin, likely related to acute injuries, while four (18.2%) patients had atraumatic or degenerative tears, often associated with age-related degeneration or chronic overuse. Lifestyle factors, such as alcohol use, were reported by four (18.2%) patients, and five (22.7%) patients were identified as smokers, both of which have been previously linked to impaired tendon healing and vascularity.

Over the 12-month study period, 21 patients were available for follow-up at three months and six months post surgery, and their outcome was assessed clinically and radiologically with an MRI. The ASES score was assessed at day zero (preoperative), three months, and at the end of six months. The score showed an increasing trend in all 21 (100%) patients at the end of six months.

The mean ASES score increased from a minimum of 28.12 at the day zero time-point, 62.89 at three months, and rose to a maximum of 82.68 at the six months time-point (Table 3). This change was statistically significant: Friedman test: N = 21, χ2 = 42.0, p < 0.001; degree of freedom (df) = 2; effect size: Kendall's W (Kendall's coefficient of concordance) = 1.000 (perfect agreement). Hence, this shows that all patients reported improved clinical outcomes at the end of six months, and were statistically significant.

**Table 3:** Variation of ASES scores from day zero to three and six months. ASES: American Shoulder and Elbow Surgeons.

Time-point	ASES score			Friedman test	
	Mean (SD)	Median (IQR)	Range	χ2	P-value
Day 0	28.12 (8.38)	28.33 (6.67)	13.33 - 51.66	42.0	<0.001
3 Months	62.89 (8.63)	61.66 (10.00)	40.00 - 83.33		
6 Months	82.68 (6.67)	82.00 (3.34)	58.00 - 90.00		

Preoperative MRI scans revealed that 14 (63.6%) patients had isolated supraspinatus tears, while eight (36.4%) patients had involvement of both the supraspinatus and infraspinatus tendons. The majority (19 patients, 86.4%) had complete thickness tears, and the remaining three patients (13.6%) had near-complete tears.

At six months postoperatively, all patients underwent a follow-up MRI to assess the integrity of the tendon repair using the Sugaya classification. Radiological outcomes of 20 out of 21 (95.2%) patients demonstrated tendon healing, classified as Sugaya type I, II, or III.

**Figure 4:**
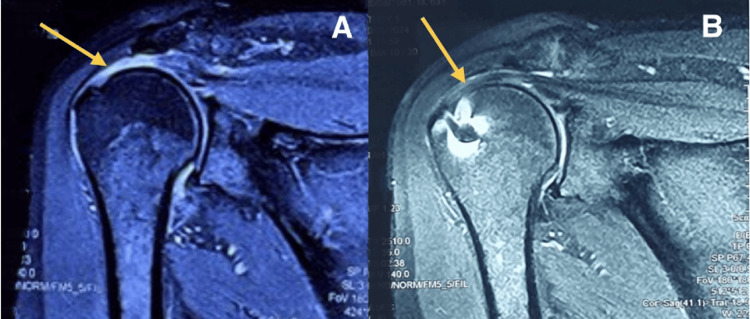
(A) Preoperative MRI of a patient with coronal section showing T2W/STIR hyperintensity (arrow) with avulsed and retracted supraspinatus tendon from the footprint. (B) MRI of the same patient done at six months postoperatively, showing tendon healing with sufficient thickness (Sugaya type I), as shown with the arrow. T2W/STIR: T2-weighted short-tau inversion recovery imaging.

One such MRI of a patient is shown in Figure 4A, which shows an avulsed and retracted supraspinatus tendon from the footprint. A follow-up MRI done six months post surgery (Figure 4B) demonstrates good healing of the supraspinatus tendon (Sugaya type I).

One patient (4.7%) had a minor discontinuity in the supraspinatus tendon seen on more than one MRI slice (Sugaya type IV) and was thus categorized as a “radiological failure.” Despite this, the patient still showed significant clinical improvement. 

Further analysis was conducted to evaluate whether patient demographics (age, sex, side of involvement, etiology, alcohol intake, and smoking) had any statistically significant association with either radiological outcome (tendon integrity on MRI) or clinical outcome (improvement in ASES scores). No statistically significant correlations were found, indicating that in this study cohort, these variables did not appear to influence healing or recovery. However, this may be due to the limited sample size, and further studies with larger populations are warranted to investigate these potential associations more robustly.

One patient developed postoperative adhesive capsulitis, which was managed successfully with conservative treatment, including physiotherapy. The patient eventually regained a satisfactory range of motion and reported functional improvement.

## Discussion

At follow-up after a minimum of six months, most patients showed good tendon healing on imaging, and functional recovery was significant, as reflected by a marked improvement in the ASES scores. These results indicate that this technique may strike a good balance between mechanical stability and biological healing support.

The average age of our patients was 55.2 years, which is a bit younger than what Dierckman et al. reported, which was 60 years for successful repairs and 66 years for failed ones [7]. While the age difference was not statistically significant, it is worth noting that age has long been linked to healing potential. Older patients often face age-related changes in tendon quality and blood supply, which may affect outcomes. Our relatively younger group might partly be the cause of better outcomes. We also saw a male predominance, which lines up with earlier studies. However, similar to findings by Dierckman and colleagues, we did not find any significant link between gender and outcome, either clinically or radiologically. This suggests that sex alone probably does not affect healing after rotator cuff surgery.

A majority of patients, i.e., 16 (72.7%), had tears on their dominant side, and many of them showed improvement post surgery. Still, this trend was not statistically significant. While one could speculate that using the dominant arm more might influence both healing and how symptoms are perceived, our data did not clearly support this.

Trauma stood out as the most common cause of rotator cuff tears in our group, accounting for 18 (81.8%) cases. Patients with traumatic tears tended to do better, both clinically and on imaging. This echoes the findings by Gutman et al. [8], who showed that early surgical repair in traumatic cases leads to better function. Even though this trend in our study was not statistically significant, it does suggest that traumatic tears might respond more favorably to surgery, likely due to tear patterns and more active biological healing.

Lifestyle factors like smoking and alcohol use were not significantly linked to poorer outcomes in our cohort. This is somewhat surprising given that both have been associated with impaired healing in a study by Passaretti et al. [9]. However, our small sample size may have limited our ability to detect such effects.

In terms of recovery, ASES scores improved dramatically from an average of 28.1 before surgery to 82.7 at six months (p < 0.001). What is particularly noteworthy is that 20 (95.2%) patients who improved clinically also showed tendon healing on imaging, reinforcing the connection between structure and function. Still, it is important to remember that this is not always the case. Russell et al. [10] noted in a meta-analysis that healing on imaging does not always guarantee pain relief or better function, which highlights the importance of focusing on how patients actually feel and function, not just what imaging shows.

Regarding surgical technique, single-row repairs have historically been considered inferior to double-row repairs in terms of footprint coverage and healing. However, a systematic review by Longo et al. [11] reported a re-tear rate of 14.5% for single-row repairs. Moreover, a meta-analysis by Ajrawat et al. [12] showed that bone marrow stimulation significantly reduced re-tear rates, from 31.8% down to 18.4%. This supports our belief that marrow venting adds real biological value to rotator cuff repair. Bone marrow stimulation has demonstrated a significant reduction in re-tear rates compared to repair alone, but shows similar short-term functional outcomes in terms of range of motion and pain. This has been confirmed in a systematic review and meta-analysis performed by Zhang et al. [13].

A study by Mahar et al. [14] on 18 bovine specimens showed that double-row repair did not show a biomechanical advantage compared to single-row repair on cyclical loading. With that in mind, though double-row repair theoretically offers a potentially larger footprint, it must be balanced against increased surgical time, complexity, and higher cost.

Similar to our study, Arroyo et al. reported more than 90% healing rates demonstrated on postoperative MRI and excellent patient-reported outcomes [15]. The only difference was the usage of triple-loaded anchors instead of double-loaded anchors in our study, and their technique, famously known as the “SCOI (Southern California Orthopedic Institute) row technique,” has shown promising results and good outcomes over time.

The use of platelet-rich plasma has also been studied as a technique for biological augmentation. It has not shown statistically significant differences in overall gain in outcome scores or re-tear rates between treatment groups, as concluded in a systematic review and meta-analysis by Warth et al. [16].

## Conclusions

To sum up, our findings suggest that a simple, medially based single-row technique when paired with bone marrow vents can be an effective option for managing medium to large rotator cuff tears, as evident with our clinical and radiological outcomes at the end of six months, which is also comparable to historical controls. While we observed several promising trends in other studies, such as better outcomes with traumatic tears or younger age, we could not derive a statistically significant correlation with healing rates, likely due to our small sample size. The lack of a control group is another limitation of the study. Still, the direction of these findings is encouraging and has provided us with enough evidence for future research in this field.

Looking ahead, larger studies with longer follow-up will help us better understand which factors really drive outcomes. But for now, our results add to the growing body of evidence that combining solid surgical mechanics with biological stimulation can lead to a meaningful and functional recovery with reduced chances of failure.
